# Life satisfaction in Norwegian medical doctors: a 15-year longitudinal study of work-related predictors

**DOI:** 10.1186/s12913-019-4599-7

**Published:** 2019-10-22

**Authors:** Javed Iqbal Mahmood, Kjersti Støen Grotmol, Martin Tesli, Torbjørn Moum, Ole Andreassen, Reidar Tyssen

**Affiliations:** 1 Department of Behavioural Sciences in Medicine, Institute of Basic Medical Sciences, Faculty of Medicine, University of Oslo, PO. Box 1111 Blindern, N-0317 Oslo, Norway; 2 Regional Advisory Unit on Palliative Care, Department of Oncology, Oslo University Hospital, Oslo, Norway; 3 NORMENT, KG Jebsen Centre for Psychosis Research, Division of Mental Health and Addiction, Oslo University Hospital & Institute of Clinical Medicine, University of Oslo, P.O. Box 4956 Nydalen, N-0424 Oslo, Norway

**Keywords:** Life satisfaction, Work-related stress, Doctors, Physicians, Cohort study, Longitudinal study

## Abstract

**Background:**

Despite many recent studies on burn-out and dissatisfaction among American medical doctors, less is known about doctors in the Scandinavian public health service. The aims of this study were to analyse long-term work-related predictors of life satisfaction among established doctors in Norway and to identify predictors in a subgroup of doctors who reported a decline in life satisfaction.

**Methods:**

Two nationwide cohorts of doctors (*n* = 1052), who graduated medical school 6 years apart, were surveyed at graduation from medical school (T1, 1993/94 and 1999), and 4 (T2), 10 (T3), and 15 (T4) years later. Work-related predictors of life satisfaction (three items) obtained at T2 to T4 were analysed. Individual and lifestyle confounders were controlled for using mixed-models repeated-measures analyses, and logistic regression analyses were applied to identify predictors of the decrease in life satisfaction.

**Results:**

Ninety per cent (947/1052) responded at least once, and 42% (450/1052) responded at all four times. Work-related predictors of higher life satisfaction in the adjusted model were work–home stress (β = − 0.20, 95% confidence interval [CI] = − 0.25 to − 0.16, *p* < 0.001), perceived job demands (β = − 0.10, CI = − 0.15 to − 0.05, *p* < 0.001), and colleague support (β = 0.05, CI = 0.04 to 0.07, *p* < 0.001). The new adjusted individual predictors that we identified included female gender, reality weakness trait, and problematic drinking behaviour. Neuroticism trait and low colleague support predicted a decrease in life satisfaction.

**Conclusions:**

Work–home stress, perceived job demands, and colleague support were the most important predictors of life satisfaction related to doctors’ work. When personality traits were controlled for, female doctors were more satisfied with their life than male doctors. These findings suggest that improving work-related factors with targeted interventions, including a supportive work environment, may increase life satisfaction among doctors.

## Background

Increased burn-out and decreased well-being have been reported for American doctors in the past decade, and lower life satisfaction among doctors can negatively affect the quality of patient care [1, 2]. Representative longitudinal studies are needed to identify long-term work-related predictors of low life satisfaction that may be targeted by organizational interventions to increase doctors’ well-being [3].

Although some cohort studies have shown that doctors report more work-related stress and emotional distress than others in the population, most evidence has been about doctors early in their career [4–7]. Less is known about the predictors of positive psychological outcomes, life satisfaction, and overall well-being in established doctors beyond the first postgraduate years [8]. In particular, few long-term longitudinal studies have controlled for the effects of possible confounders, such as individual factors, personality, lifestyle factors, and life stress outside of work, including negative life events, on work-related pressure [9]. Our unique 15-year follow-up study of two nationwide cohorts of 1052 Norwegian doctors has been designed for this purpose. Because the cohorts graduated medical school 6 years apart, it is possible to study the effects of recent trends induced by health reforms.

In our previous study of work-related factors, we found that work stress is important to life satisfaction early in the career of Norwegian doctors [10]. However, that study did not identify the types of work-related pressures that affect doctors or the relative effects of these factors. Reliable data reflecting the effects of long-term stresses on doctors’ work can be identified only by long follow-ups, and such studies are warranted [11]. Possible work-related factors include time pressure [12], emotionally demanding patient work [13], and work–home stress [14]. The latter has been found to also predict emotional exhaustion in doctors beyond the first years of their career [14]. Work–home stress seems to be more important among women early in their career [14–16], but few studies have included established career doctors. Doctors’ long hours [17] and lack of sleep should be included in studies of doctors’ well-being [18]. According to Karasek and Theorell’s demand–control–support model, low decision latitude (excessive work demands at the expense of autonomy at work) and lack of social support can lead to stress, mental health problems, and cardiovascular disease [19, 20]. However, these factors have been studied less among doctors than among other health care workers [21].

During the past two decades, the health care system in Norway has undergone major changes following three important reforms. (I) “The Regular General Practitioner scheme” in 2001, a list-patient system whereby all inhabitants in Norway have their assigned general practitioner and, (II) “The Hospital Reform” in 2002 transferred the ownership of hospitals and specialist health services from the county to the state level, and this reform aimed at a better effectivity (cost-control) and quality of services, being based on the principles of New Public Management. (III) “The Coordination Reform” from 2012 intended to develop more integrated patient pathways, by improving the collaboration between specialists (secondary) and municipal (primary) health care levels, and more prevention. By studying two Norwegian cohorts that graduated 6 years apart, we reasoned that we could identify putative effects of the reforms on work-related factors because the younger cohort had been influenced by these changes over an extended period of time.

In the statistical analyses in this study, we controlled for individual factors that have been linked to life satisfaction, such as partner support [22], having children [23], social support [23, 24], physical activity [25], and religious activity [26]. The personality trait of neuroticism may be associated with a low sense of well-being [10, 24]. Problematic drinking behaviours may also be linked to poor life satisfaction [27]. Over the years, many doctors experience negative life events that may impair their well-being [24].

In contrast to our previous study of early career doctors [10], in the present study, we used a more reliable three-item measure of life satisfaction and repeated-measures statistical analyses of three time periods up to 15 years after graduation, rather than relying on a single regression and a single item measure of life satisfaction [10]. In addition, we have included the personality trait of reality weakness (which is linked to severe personality disorders, such as borderline and schizotypal) because we believe that this may affect the well-being of doctors. Because of the increasing proportion of female doctors in Norway, we attempted to identify gender differences in the predictors of life satisfaction in the study population. In this study, we addressed four specific research questions.

1. What is the trend of life satisfaction among Norwegian doctors during the 15 years after graduation?
2. What work-related factors predict life satisfaction after adjusting for possible confounding individual and lifestyle factors?
3. Are there any gender and/or cohort effects on the predictors identified?
4. What factors predict a decrease in life satisfaction at the individual level over a 15-year follow-up period?

## Methods

### NORDOC sample (the longitudinal study of Norwegian medical students and doctors)

Two nationwide cohorts of medical students (Medical Student Cohort and Young Doctor Cohort) who graduated 6 years apart were followed for 15 years after graduation [28, 29]. The cohorts were merged (*n* = 1052), and the present data were obtained at four time points: T1, final year of medical school (data collected in 1993/94 and 1999, *n* = 892 and 1052, respectively, 85% of the eligible sample); T2, 4 years after graduation (data collected in 1998 and 2003, *n* = 780 and 1052, respectively, 74% of the eligible sample); T3, 10 years after graduation (data collected in 2003 and 2008, *n* = 708 and 1052, respectively, 67% of the eligible sample); and T4, 15 years after graduation (data collected in 2008 and 2014, *n* = 598 and 1052, respectively, 57% of the eligible sample). Ninety per cent (947/1052) responded at least once, and of those who responded prospectively, 42% (450/1052) responded at all four time points. Fifty-nine per cent were women (264), and 41% (186) were men.

### Measures

#### Dependent variable

Life satisfaction was measured at T2, T3, and T4 using three items. Item 1 included the question, “When you think about your life today, would you say that you are by and large satisfied with life, or are you mostly dissatisfied?” The response alternatives ranged from (1) extremely dissatisfied to (7) very satisfied. This and similar items have been validated in previous studies [10, 30, 31]. Item 2 included the question, “To what extent are you satisfied with your daily life situation?” The response alternatives ranged from (1) very dissatisfied to (4) very satisfied. Item 3 included the question, “Would you describe yourself mostly as …?” with response alternatives ranging from (1) not happy at all to (5) very happy. A factor analysis of these three items at T2 yielded an unequivocal unidimensional solution from which the factor score coefficients were multiplied by their respective constituent items (raw scores) and summed to create a simple additive scale. The factor score coefficients derived at T2 were also used at T3 and T4 (i.e., without standardizing the raw scores before multiplying and summing). This procedure allowed us to apply a mixed-model repeated-measures approach in our statistical analyses. Cronbach’s α values for this scale were 0.83, 0.83, and 0.82 at T2, T3, and T4, respectively.

#### Predictor variables

*Perceived job stress* was measured from T2 to T4 using a modified version [32] of the Cooper Job Stress Questionnaire [33]. Each item was measured on a five-point scale (1 = no stress, 5 = a lot of stress). A factor analysis performed at T2 [16] identified four dimensions: emotional pressure (eight items, α = 0.83), time pressure (six items, α = 0.71), fear of complaints/criticism (seven items, α = 0.75), and work–home interference (three items, α = 0.86). The mean item score of each dimension was used as a measure of the level of perceived job stress, and this variable has been previously validated [29].

Two other workload measures were included from T2 to T4: average *number of working hours per week* and average *number of hours asleep when on call* [29].

Psychosocial factors at work such as *perceived job demands* and *autonomy* were measured from T2 to T4 using 10 statements that have been described and validated elsewhere [34]. A factor analysis of these 10 items at T2 identified two factors: perceived job demands (eight items, α = 0.82) and autonomy (two items, α = 0.78). The summed score for each factor was used in the analysis. Examples of questions or statements to assess perceived job demands were: “Do you sometimes have so much to do that your work situation becomes hurried and taxing, and if so, how often?” and “You work under unacceptable pressure”. These two variables resemble Karasek’s demand–control variables in the General Nordic Questionnaire for Psychological and Social Factors at Work (QPS Nordic) [35], which was implemented in the Medical Student Cohort at T4 in 2014.

*Colleague support* was measured from T2 to T4 by the following two items: “To what degree are you taken care of by your colleagues?” and “To what degree do you enjoy working with your colleagues?” Each item was measured on a seven-point scale from 1 = not at all to 7 = to a very high degree. The summed score was used as a measure of the level of colleague support. Cronbach’s α values were 0.80, 0.83, and 0.85 for T2, T3, and T4, respectively. This variable has been previously validated [14, 16].

*Age* was measured as a continuous variable.

*Having children* was measured from T2 to T4 by the question, “How many children do you have?” and the response categories are described elsewhere [28]. We dichotomized this variable as no children = 0 and ≥ 1 children = 1.

*Married/cohabitant* status was measured from T2 to T4 by one question about current marital status, which was scored as 0 = unmarried, separated, widowed/widowed, or divorced, and 1 = married or cohabiting [10].

*Perceived social support* was measured at T2 to T4 by five questions about the degree of appreciation by close friends, presence of warm and caring confidants, degree of affiliation with groups such as the neighbourhood, political organizations, or church, and the support anticipated if they should fall ill. All items were scored using five response categories, which were then summed; a higher total score indicated a higher level of experienced support [10].

Frequency of physical activity was measured at T2 to T4 by the question, “Do you usually work out or do physical exercise such as jogging, cycling, swimming, etc.?” using response categories that have been described and validated elsewhere [10].

*Religious activity* was measured at T2 to T4 by one item that asked about the frequency of engagement in any kind of religious activity, which has been described and validated in previous studies [28, 29]. We dichotomized this variable into 0 = no religious activity and 1 = participation in any type of religious activity.

*Use of alcohol to cope with tension* was measured at T2 to T4 by the question, “When you feel worried, tense, or nervous, do you ever drink alcoholic beverages to help you handle things?” The response alternatives were: never, seldom, occasionally, and often. The variable was dichotomized at 1 = yes (any frequency) and 0 = never. This variable has been validated in surveys conducted in Norway [28, 29, 36] and in the US [37].

*Hazardous drinking* was measured from T2 to T4 with a modified nine-item version of the Alcohol Use Disorder Identification Test. This instrument has been described and validated in a previous study [29].

*Personality traits* were measured using the 36-item version of Torgersen’s Basic Character Inventory (BCI), which assesses the four personality trait dimensions: vulnerability, intensity, control, and reality weakness. The first three dimensions resemble Eysenck’s Giant Three [38]: neuroticism, extraversion, and conscientiousness, respectively, and these terms are used here. Reality weakness is a deviant trait involving perceptions and ideation on the borderline between reality and fantasy, and it measures chronic illusions, paranoid traits, and problems with identity, insecurity, and relationships, traits that are linked to severe personality disorders, such as borderline and schizotypal personality disorders [39]. Examples of statements indicating reality weakness items are “Sometimes I feel I am not myself”, “I experience myself as being totally different at different points in time”, and “Sometimes I seem to live in a fog”. The latter two items have been associated with aggravation of suicidal ideation [40]. Reality weakness captures both impaired “self” or “identity integration” and interpersonal functioning. It overlaps with some of the personality disorder trait domains in Section III of the Diagnostic and Statistical Manual of Mental Disorders (fifth edition), and it has shown predictive validity for identifying emotional disturbance in physicians [41]. Each dimension ranges from 0 = low to 9 = high and is based on a total score of the answers to nine questions, each with a dichotomous response (agree or do not agree). The 36-item version of the BCI has been widely used in studies of Norwegian medical students and doctors [28, 29, 32, 42].

*Life events* during the past year were measured from T2 to T4 by 13 items that have been described and validated in several studies of the two cohorts [40, 43]. All items were coded 0 or 1, and the summed score of all items (including both positive and negative life events) was used in the analysis.

Descriptions of all independent variables are shown in Table 1.

**Table 1:** Description of the independent and dependent variables (outcome variable) measured at the four times points

Predictor variables	T1 Final year of graduation	T2 4 years after graduation	T3 10 years after graduation	T4 15 years after graduation
Age, years (continuous)	28 (2.83)			
Gender, female, %	56			
Having children, %		55	82	91
Married/cohabitating, %		78	83	87
Perceived social support		14.72 (6.24)	20.38 (2.89)	20.32 (2.85)
Physical activity		1.80 (1.01)	1.89 (1.01)	2.17 (1.02)
Religious activity, %		27	26	28
Use of alcohol to cope with tension, %		8	11	12
Hazardous drinking, %		13	13	15
Neuroticism	3.52 (2.23)			
Extraversion	5.46 (2.36)			
Reality weakness	1.44 (1.59)			
Conscientiousness	3.09 (2.07)			
Life events		1.05 (1.19)	0.72 (0.90)	0.55 (0.88)
Contextual work-related variables				
Emotional pressure		2.07 (0.65)	1.86 (0.58)	1.79 (0.53)
Time pressure		2.41 (0.69)	2.25 (0.68)	2.16 (0.67)
Fear of complaints and criticism		2.11 (0.67)	1.93 (0.65)	1.80 (0.56)
Work–home interference		2.46 (1.00)	2.42 (1.00)	2.33 (0.99)
Number of hours at work per week		45.29 (9.91)	42.99 (8.89)	42.21 (10.96)
Number of hours asleep when on call		4.52 (2.79)	5.38 (6.36)	6.41 (7.14)
Perceived job demands		1.96 (0.96)	1.92 (0.99)	1.92 (0.97)
Autonomy		2.28 (0.71)	2.19 (0.70)	2.22 (0.68)
Colleague support		10.00 (2.42)	9.76 (2.36)	10.13 (2.38)
Outcome variable				
Life satisfaction (3 items)		4.55 (0.82)	4.60 (0.81)	4.62 (0.81)

Data are shown as percentages or means (SD) where not otherwise specified

### Statistical analysis

To analyse life satisfaction (three-item scale), we used linear mixed models with a repeated unstructured covariance matrix and maximum-likelihood estimator (or algorithm) for all predictor variables. This was a longitudinal study (panel study) in which the same individuals were followed and assessed repeatedly over four time intervals. Applying linear mixed models made the estimates of effect parameters more robust. This method takes into account the related associations between measures for individuals, uses longitudinal samples more efficiently, is less vulnerable to missing data, and has greater statistical power because it implements a covariance matrix.

All independent variables, including the time variable, were treated as fixed effects. First, we performed unadjusted analyses of all predictor variables for life satisfaction from T2 to T4 as the dependent variable, and these variables were then entered into two models in the adjusted analyses (see Table 2). In Model 1 (adjusted model), only significant predictor (or individual) variables in the univariate analysis were entered. In Model 2 (adjusted and final model), significant variables from Model 1, significant univariate contextual variables from block 2, and the time variable were entered. In all analyses, we controlled for age, gender, and cohort. Only variables with *p* < 0.05 were considered to be significant in the final adjusted Model 2. Additional interaction analyses using both gender and cohort were performed to identify any gender and/or cohort differences in the adjusted predictor effects.

**Table 2:** Predictors of life satisfaction using linear mixed models

Unadjusted					Adjusted							
					Model 1 (*n* = 633)				Model 2 (*n* = 590)^a^			
	b	t	95% CI	*p*-value	b	t	95% CI	*p*-value	b	t	95% CI	*p*-value
Block 1: Individual and lifestyle factors												
Age (continuous)	− 0.02	−2.54	− 0.04– –0.005	0.011	−0.007	− 0.81	− 0.02–0.01	0.418	− 0.009	− 1.07	− 0.02–0.007	0.281
Gender, female	0.03	0.68	−0.06–0.12	0.494	0.11	2.33	0.01–0.21	0.020	0.09	2.03	0.003–0.18	0.043
Having children	0.12	3.21	0.05–0.20	0.001	0.01	0.35	−0.07–0.10	0.722				
Married/cohabiting	0.31	6.94	0.22–0.40	< 0.001	0.33	6.44	0.23–0.44	< 0.001	0.46	9.16	0.36–0.56	< 0.001
Perceived social support	0.02	7.78	0.01–0.02	< 0.001	0.01	3.10	0.004–0.01	0.002	0.02	4.52	0.01 − 0.03	< 0.001
Physical activity	0.13	8.25	0.10–0.16	< 0.001	0.12	6.70	0.08–0.15	< 0.001	0.08	4.49	0.04–0.12	< 0.001
Religious activity	0.05	1.25	–0.03–0.14	0.209								
Use of alcohol to cope with tension	−0.44	−7.94	−0.55– −0.33	< 0.001	–0.33	−5.45	− 0.46– –0.21	< 0.001	−0.24	−3.89	− 0.37– –0.12	< 0.001
Hazardous drinking	−0.26	−5.17	− 0.36– − 0.16	< 0.001	–0.16	−2.95	− 0.27– –0.05	0.003	− 0.16	−2.93	−0.27– –0.05	0.003
Neuroticism	−0.07	−6.89	−0.10– − 0.05	< 0.001	–0.05	− 4.76	− 0.08– –0.03	< 0.001	− 0.04	− 3.66	−0.06– –0.01	< 0.001
Extraversion	0.04	4.03	0.02–0.06	< 0.001	0.02	2.39	0.004–0.04	0.017	0.01	1.31	−0.006–0.03	0.188
Reality weakness	−0.10	−6.46	−0.13– –0.07	< 0.001	− 0.05	−3.31	− 0.09– –0.02	0.001	− 0.03	−2.18	−0.06– –0.003	0.029
Conscientiousness	−0.01	−1.24	−0.04–0.009	0.214								
Medical student cohort	0.11	2.46	0.02–0.21	0.014	0.10	2.05	0.004–0.20	0.041	−0.04	−0.79	−0.14–0.06	0.425
Life events	−0.12	−7.96	−0.14– – 0.09	< 0.001	− 0.07	−4.27	− 0.10– –0.03	< 0.001	− 0.08	− 4.77	−0.11– –0.04	< 0.001
Block 2: Contextual work-related factors												
Emotional pressure	−0.30	−10.25	−0.36– –0.24	< 0.001					−0.07	−1.70	−0.16–0.01	0.089
Time pressure	−0.34	−13.85	−0.39– –0.29	< 0.001					0.006	0.16	−0.07–0.09	0.873
Fear of complaints and criticism	−0.22	−7.96	−0.28– –0.17	< 0.001					0.04	1.02	−0.03–0.11	0.305
Work–home interference	−0.29	−18.34	−0.32– –0.26	< 0.001					−0.20	− 8.67	− 0.25– –0.16	< 0.001
Number of hours at work per week	−0.004	−2.70	− 0.008– − 0.001	0.007					0.002	1.25	–0.001–0.006	0.210
Number of hours asleep when on call	−0.0007	− 0.18	− 0.008–0.007	0.851								
Perceived job demands	−0.24	−14.18	−0.27– –0.20	< 0.001					−0.10	−4.24	−0.15– –0.05	< 0.001
Autonomy	0.08	3.73	0.04–0.13	< 0.001					−0.04	−1.58	−0.09–0.01	0.113
Colleague support	0.08	12.30	0.07–0.09	< 0.001					0.05	7.02	0.04–0.07	< 0.001

b = unstandardized regression coefficients; t = *t* value; CI = confidence interval

^a^Time T2 was used as a reference in final Model 2 because most of the individual factors and all of the contextual and lifestyle factors were measured repeatedly at T2–T4

We also analysed the dependent variable from T2 to T4 with three clusters: (I) the group with increasing life satisfaction (*n* = 71); (II) the group with stable high life satisfaction (*n* = 331); and (III) the group with a decline in life satisfaction (*n* = 87). Logistic regression analyses were performed on group (III) to identify which factors predicted a decrease in life satisfaction.

## Results

### Course of life satisfaction in doctors (longitudinal sample)

The overall mean values on the life satisfaction additive scale remained consistently at about the same level: 4.55 (SD = 0.82) at T2, 4.60 (SD = 0.81) at T3, and 4.62 (SD = 0.81) at T4. There were no significant gender differences.

### Predictors of life satisfaction in doctors

The unadjusted predictor variables for life satisfaction are shown in Table 2. The adjusted predictors in the final model were (Table 2, Model 2): low work–home stress (*p* < 0.001), low perceived job demands (*p* < 0.001), colleague support (*p* < 0.001), female gender (*p* = 0.043), being married/cohabitating (*p* < 0.001), perceived social support (*p* < 0.001), physical activity (*p* < 0.001), no use of alcohol to cope with tension (*p* < 0.001), no hazardous drinking (*p* = 0.003), low neuroticism trait (*p* < 0.001), low reality weakness trait (*p* = 0.029), and few life events (*p* < 0.001). The predictors that contributed most were low levels of work–home stress (*t* = − 8.67), colleague support (*t* = 7.02), and being married/cohabitating (*t* = 9.16). Model 2 accounted for 21% of the total explained variance. Post hoc univariate linear mixed-model analyses showed that only negative life events (divorce/separation/broken relationship, serious disease/accident/hospital admission, etc.) predicted lower life satisfaction.

The percentages of primary care physicians (GPs) and hospital specialists were 22% (*n* = 105) and 78% (*n* = 375), respectively. The level of life satisfaction did not differ significantly between primary care physicians and specialists working in hospitals; this lack of difference ruled out possible confounding by this independent variable.

Female gender became significant in Model 1 when the personality trait of neuroticism was entered because the very weak uncontrolled positive effect of female gender was “suppressed” by the combination of a relatively positive correlation between female gender and neuroticism (Pearson’s r [r] = 0.24) and a negative correlation (r = − 0.22) between neuroticism and life satisfaction.

### Interaction effects with gender and cohort

There was a significant positive interaction between male gender and the use of alcohol to cope (β = 0.28, CI = 0.06 to 0.50, *p* = 0.011), which indicates that the negative effect of the use of alcohol to cope was significantly stronger among males. In addition, the negative effects of neuroticism (β = 0.04, CI = 0.004 to 0.09, *p* = 0.032) and perceived job demands (β = 0.09, CI = 0.02 to 0.16, *p* = 0.005) were significantly stronger among men than women (interactions not shown in Table 2).

There was a cohort effect of work–home stress on life satisfaction (β = − 0.07, CI = − 0.14 to − 0.01, *p* = 0.016), which indicates a significantly stronger effect among the younger cohort (graduated in 1999) of doctors. Additional analyses showed that the young doctor cohort reported the highest levels on all four perceived job stress dimensions. No significant interactions were found between cohort and perceived job demands or colleague support.

### Predictors of a decrease in life satisfaction

Adjusted significant predictors of a decrease in life satisfaction (using both the stable group and the group with increasing life satisfaction as a reference) were neuroticism (odds ratio [OR] = 1.25, 95% CI = 1.07 to 1.47, *p* = 0.004) and colleague support measured at T2 (OR = 0.84, 95% CI = 0.73 to 0.97, *p* = 0.021).

## Discussion

A major finding of this study is that low work–home stress, low perceived job demands, and high colleague support were independent predictors of life satisfaction among Norwegian doctors. There was a cohort effect on all job stress dimensions, involving primarily work–home stress, which suggests that this type of stress has increased over the past decade in Norwegian doctors. The predictors of a decrease in life satisfaction over the 15 years were neuroticism trait and low colleague support.

The course of life satisfaction in doctors remained stable from years 4 to 15 after medical school. This is consistent with an earlier longitudinal study (1994–2002) of Norwegian doctors [44]. American and British studies have shown relatively high levels of stress, dissatisfaction, and depression among resident doctors [7, 45, 46], which is similar to what has been found among Norwegian doctors [16, 32, 47]. The present study suggests that certain aspects of doctors’ work impose a heavy toll on doctors’ well-being beyond the first stressful period of their career.

Scandinavian work life is well regulated, and we believe that the negative work-related factors identified in Scandinavia may be even more detrimental in countries with less regulation of work. This means that the factors identified, such as work–home stress and perceived job demands, may have an even more deleterious effect in the private health care system such as in the US. American studies have concluded that work–home stress explains much of the variance in both burn-out and depressive symptoms [48, 49]. Burn-out is a syndrome characterized by emotional exhaustion, depersonalization, and a low sense of personal accomplishment [50]. Depression is a mental disorder characterized by persistent sadness (low mood) and loss of interest in activities that a person normally enjoys, and is often accompanied by the inability to perform activities of daily life [51]. Scales measuring burn-out have been poorly validated with respect to a clinically valid cut-off, whereas scales measuring depressive disorder have been clinically validated [52]. Burn-out and depression may be related to each other in terms of emotional exhaustion, but they differ because, operationally, burn-out by definition is related to experiences at the workplace and job stress. Depression, however, is often related to negative life events outside of work, such as separation from a partner or having a severe or chronic physical disease.

Our findings are consistent with studies showing that greater colleague support is linked to a better sense of well-being [8, 53]. Thus, support from peers and a good working environment may buffer some of the stressors related to doctors’ work [16]. The present study is the first to show that low perceived support from colleagues is independently related to a decline in the life satisfaction of some doctors over 15 years. Another important work-related predictor was that of perceived job demands. We argue that low life satisfaction among doctors probably pertains to specific demands related to their work life. Such demands may relate to being responsible for the medical care and treatment of individual patients with severe disease or impending death under time pressure, as well as high expectations about increased productivity in many hospitals. This ethical, legal, and often emotionally draining responsibility is a demand that is relatively unique to the medical profession and is pertinent in the Scandinavian public health system.

The negative effect of perceived job demands was strongest for men, and there was no gender difference in the effects of work–home stress, which is contrary to what was reported in a US study [2]. There may be several reasons for this. First, demanding patients may be easier to treat for women, who are assumed to be better communicators [54]. Second, women more often choose part-time work and may give up a difficult and straining medical career because of family demands [16]. Third, Scandinavian social benefits have reduced the pressures on women related to childbirth and childcare, and it has been shown that women doctors receive more support from colleagues [16]. In a previous study, an important adjusted predictor of work–home stress has been shown to be the number of children [16]. In the present study there was indeed a significant but rather weak association between work–home stress and number of children (r = 0.10), but the overall effect of number of children on life satisfaction is positive and weak (r = 0.09). Number of children did not confound the negative impact of work–home stress on life satisfaction since both variables retain their effects when simultaneously controlled.

Work–home stress had a stronger negative impact on life satisfaction in the youngest cohort of doctors. These doctors experienced the health service reforms mentioned in the introduction in the most stressful years of their early career. There is evidence that the Hospital Reform in 2002 and the Coordination Reform in 2012 have increased pressures on both hospital physicians and general practitioners in Norway [55, 56].

With respect to interventions, organizational measures that target work–home stress and overly high job demands on doctors may improve their life satisfaction and possibly reduce the prevalence of burn-out and depression [3]. The importance of adequate colleague support, such as better communication between older and younger doctors, as well as available substitutes when ill, should also be considered [57]. Organizational efforts may be important for ensuring adequate colleague support and may help to limit or prevent the reduction in life satisfaction in later years of doctors’ careers.

Female Norwegian doctors were more satisfied with life only in the model that adjusted for individual factors such as personality. Studies have found US female doctors to be less satisfied with their lives and to report higher levels of depression [12, 46], and these findings are consistent with those of a Spanish study of hospital staff [58]. A Swiss study has shown female doctors to be more satisfied than their male counterparts [59], and our data are consistent with this finding. Therefore, there may be cross-national and cultural differences here.

We found few differences between male and female Norwegian doctors with respect to levels of overall well-being and emotional distress, which may reflect the relatively equal gender roles and regulated work life in Scandinavia [14, 47]. In Norway, the female-to-male ratio in medical students has increased over the past decades. The reasons for this may be a loss of status and economic benefits in a career in medicine compared with other occupations as perceived by young men, but this needs to be confirmed in further studies. Interestingly, the gender effects in general were found to be weaker or much smaller than expected. Nevertheless, it could be argued that the growing proportion of women in the medical profession may lead to a “feminization” of the profession, which may influence the self-understanding, norms, and frames of reference among both male and female doctors and hence their responses. Testing this hypothesis would require both longitudinal and cross-national data, to which we do not have access.

As expected, a number of individual predictors and lifestyle variables were linked to life satisfaction. Interestingly, neuroticism trait (or low self-esteem) was an independent predictor of life satisfaction in the sample as a whole but was also an adjusted predictor in the subgroup of doctors who experienced a reduction in life satisfaction. This trait has previously been found to predict depression and poor mental health in studies of doctors and other populations [24, 41]. Our study suggests that problematic drinking behaviours (drinking to cope and hazardous drinking) play an unhealthy role, particularly in male doctors. This may reflect the concept that drinking is a male behaviour. Men and women tend to use different means to cope with stress: men tend to use alcohol whereas women tend to use tranquillizers [60, 61]. One or our findings was that the reality weakness trait was an independent predictor of life satisfaction. This deviant trait also predicts suicidal ideation and not seeking help among Norwegian doctors [41]. The other individual predictors linked to life satisfaction such as being married/cohabiting, which is a form of structural support, and perceived social support, are consistent with the findings of other studies of doctors and the general population [23, 24, 62–64]. The importance of family life and social relationships should be emphasized. Physical activity/exercise and working less are also associated with increased well-being in doctors [12]. Few negative life events predicted higher life satisfaction in doctors, and these findings are consistent with those of other studies of doctors and the general population [24, 65].

### Strengths and limitations

The major strengths of this study include the nationwide sample, long follow-up, and the use of validated instruments. The repeated-measures analyses ensured good reliability. Life satisfaction was measured by three items instead of one item, as used in our previous study [10]. The self-reported work-related stress factors were adjusted for personality traits, which increased the credibility of the associations observed, indicating that such stress factors are experienced not only by the most vulnerable doctors.

Except for the true predictor effects of personality variables, the other observed effects were mean associations measured concurrently with the outcome. Recruitment bias at inclusion could not be investigated because we did not know the characteristics of respondents who were initially approached but who failed to respond. However, on the assumption that recruitment bias would be similar for drop-outs at each successive stage of data collection, we estimated the point estimates for variables at given time points and associations between dependent and independent variables among those who dropped out from one time point to the next. In this analysis, no parameters came even close to being statistically significant. In addition, analyses of the non-random attrition of those who responded at T1 but later dropped out (*n* = 149) showed no significant differences in the predictor variables except for a slight age difference of 1 year between the drop-outs (mean = 28.5 years, SD = 3.3) and those who remained (mean = 27.7 years, SD = 2.8). This slight age difference meant that older respondents had a greater tendency to drop out than younger ones (*p* = 0.006) [29]. Further, the NORDOC sample may be less representative of Norwegian doctors in 2014 with respect to international medical graduates and a few specialties described in detail elsewhere [29]. About 95% of our sample had a European (Caucasian) background. There was no significant association between ethnicity and our dependent variable of life satisfaction. Nor were there any statistically significant associations between ethnicity and drinking behaviours (drinking to cope and hazardous drinking). We did not have any information about the sexual orientation or gender identity of our respondents.

## Conclusions

Although working life and hours in Norway are highly regulated, work–home stress, perceived job demands, and lack of colleague support seem to have a negative effect on doctors’ well-being. Preventive measures are needed at the organizational level to reduce such types of stress among doctors. In addition, healthier coping strategies and lifestyle, and psychotherapy for those with high levels of the neuroticism trait should be considered.

## Data Availability

The datasets generated and/or analysed during the current study are not publicly available because participants’ consent was not obtained for data sharing.

## Abbreviations

CI: Confidence interval
GP: General practitioner
NORDOC: The longitudinal study of Norwegian medical students and doctors
SD: Standard deviation
T1: Final year of medical school
T2: 4 years after graduation
T3: 10 years after graduation
T4: 15 years after graduation
US: United States
β: Unstandardized regression coefficients
